# Results from a randomized controlled pilot trial of a home-visiting intervention to reduce child maltreatment

**DOI:** 10.1186/s40814-025-01659-9

**Published:** 2025-06-03

**Authors:** Joakim Finne, Anne Grete Tøge, Kjersti Stabell Wiggen, Maria Ekre, Ira Malmberg-Heimonen, Maiken Pontoppidan, Jacinthe Dion, Truls Tømmerås, Eirin Pedersen

**Affiliations:** 1 Work Research Institute, Oslo Metropolitan University, Oslo, Norway; 2 Department of Social Work, Child Welfare and Social Policy, Faculty of Social Sciences, Oslo Metropolitan University, Oslo, Norway; 3 VIVE-The Danish Center for Social Science Research, Copenhagen, Denmark; 4 Department of Psychology, Université du Québec À Trois-Rivières, Quebec, Canada; 5 The Norwegian Center for Child Behavioral Development, Oslo, Norway

**Keywords:** RCT, Child welfare services, Child maltreatment, Family partner

## Abstract

**Background:**

Child maltreatment has severe and lasting consequences, and evidence-based interventions are essential for its prevention. However, few randomized controlled trials (RCTs) have been conducted within child welfare settings in Norway. Pilot trials play an important part in assessing the acceptability and feasibility of such interventions prior to full scale evaluations. This study evaluated the acceptability and feasibility of conducting a full-scale RCT of the Family Partner home-visiting intervention, designed to reduce the risk of child maltreatment.

**Methods:**

Families from three child welfare offices in Norway with at least one child under the age of 12 were invited to participate in this pilot trial. A two-arm randomized design was used, with participants allocated in a 1:1 ratio to either the intervention or control group (*n* = 45). The intervention group received the home-visiting Family Partner intervention, while the control group received treatment as usual. A qualitative process evaluation was conducted alongside the trial, comprising 29 interviews with Family Partners, caseworkers, participating families, and other stakeholders. Statistical and qualitative analyses evaluated participant acceptability, adherence, and retention.

**Results:**

Qualitative findings indicate a high level of acceptability for the Family Partner intervention across all stakeholder groups. Adherence was strong, with no participants withdrawing consent and only two opting out of subsequent surveys. However, participant retention declined over time, with survey response rates dropping at each time point and only 42% completing the final assessment.

**Conclusions:**

This pilot trial provides preliminary evidence supporting the acceptability of the Family Partner intervention within child welfare services and highlights important considerations regarding the feasibility of conducting RCTs in this setting.

**Trial registration:**

ClinicalTrials.gov identifier NCT04957394.

## Key messages regarding feasibility

- What uncertainties existed regarding the feasibility?

There was uncertainty about whether participants in the Family Partner intervention could be randomized within child welfare settings.

- What are the key feasibility findings?

Acceptance of the Family Partner intervention within the child welfare services was evident; however, survey response rates declined significantly at each measurement point, with 42% completing the final assessment.

- What are the implications of the feasibility findings for the design of the main study?

This study demonstrates that the Family Partner intervention is feasible within child welfare contexts. However, strategies such as monetary incentives combined with personalized follow-up should be considered to improve survey response rates.

## Background

Child maltreatment is broadly defined as physical and/or emotional ill-treatment, sexual abuse, neglect, negligence, and commercial or other exploitation of children (WHO, 2022). About one in four children experience child maltreatment during childhood. Among those children, it is estimated that 18% experience physical abuse, 78% experience neglect, and 9% endure sexual abuse [11]. The consequences of child maltreatment are profound and affect multiple domains of well-being. These include mental health issues [4] such as depression [21], and increased risk of suicide attempts [2]. It is also associated with a heightened risk of later violence perpetration [18], poor academic performance [43], and health risks such as sexually transmitted infections, drug use, and risky sexual behavior [29].

In an umbrella synthesis of meta-analyses on risk factors for child maltreatment, van IJzendoorn et al. [42] identified several key risk factors, including parental experience of maltreatment during their own childhood, low socioeconomic status, dependent and aggressive parental personality, and intimate partner violence. In addition, the study found that interventions aimed at reducing child abuse potential as well as reported child maltreatment cases demonstrated overall moderate effectiveness [42]. The most commonly evaluated child maltreatment interventions using randomized controlled trials (RCT) are home visiting interventions and educational interventions [28]. Summaries of effective components in child maltreatment interventions indicate that larger effect sizes are observed for short-term interventions lasting 0–6 months, delivered by professionals, and focusing on promoting parental self-confidence. Moreover, van der Put et al. [41] found that effect sizes increased with continued follow-up of caretakers [41]. However, not all studies are equally optimistic about the effects of interventions to reduce child maltreatment. For instance, a meta-analysis by Euser et al. [15] demonstrated that the significant combined effect of the included studies disappeared after accounting for publication bias related to non-significant outcomes. Nevertheless, Euser et al. identified larger effect sizes among interventions that included parent training rather than solely providing support, as well as interventions with moderate durations and a moderate number of sessions [15]. In sum, these studies highlight the importance of evidence-based interventions, particularly those incorporating parent training and delivered over a moderate duration, as critical tools in preventing child maltreatment.

In Norway, where this study was based, the Ministry of Children and Equality has introduced amendments to the Child Welfare Act, emphasizing a shift toward greater use of family-supportive interventions. This transition reflects a broader effort to implement and evaluate new research-based interventions within child welfare services. Some interventions, such as Parent Management Training [31] and Multisystemic Therapy [32], have been evaluated through RCTs within CWS. However, counselling and parental guidance remain the most common support interventions offered to parents in Norwegian CWS. These measures typically focus on enhancing the parent–child relationship and improving parents’ caregiving skills. However, their content varies significantly and has been subject to limited research [27]. These gaps may be attributed to the complex challenges of conducting RCTs in child welfare settings, including ethical dilemmas surrounding randomization and contamination risks between study arms and uneven attrition (Finne et al.; [24, 30, 35, 36]). To address these challenges and enhance the success of large scale trials, pilot studies are recommended as a preparatory step to mitigate risks [3].

In 2021, the Family Partner intervention was developed as a support measure to prevent care takeovers within the child welfare services. The primary objective of this pilot RCT was to assess the acceptability of the Family Partner intervention for staff and caretakers and to evaluate its feasibility for a full-scale RCT. To gain a deeper contextual insight into the acceptability of the intervention from the perspectives of participants and staff, a mixed-methods design was used, incorporating both longitudinal quantitative data and qualitative data [22].

## Materials and methods

The pilot trial was designed as a prospective, parallel, RCT. Three municipalities were included in the study: Lillehammer with approximately 21,000 inhabitants, Larvik with around 48,000 inhabitants, and Øvre Eiker with about 20,000 inhabitants. These are considered medium-sized to small municipalities in the Norwegian context. In terms of service activity in each CWS office, 402 children under investigation out of 3431 children in Larvik, 213 out of 11,154 in Øvre Eiker, and 217 out of 3014 in Lillehammer [6]. The trial took place from January 2022 to February 2024. To determine eligibility, participating families were referred to child welfare services following a report of concern, in accordance with statutory provisions. They needed to meet the following criteria: (1) have at least one child under the age of 12, and (2) parents demonstrated challenges in parenting skills. These challenges included difficulties with routines, setting boundaries, parenting approaches, emotional attachment, understanding and monitoring the child’s activities, or providing practical care such as food, clothing, and hygiene. The intervention was voluntary in accordance with the Child Welfare Act.

### The family partner intervention

The Family Partner intervention is designed to reduce child maltreatment through intensive home visitation. At the core of the intervention are “Family Partners”, trained child welfare workers employed within Norwegian CWS. The primary goal of the intervention is to support at-risk parents in improving their parenting skills, self-efficacy, and trust in welfare services, while also enhancing their children’s well-being. The intervention targets families with at least one child under the age of 12 living in potentially harmful circumstances, i.e., children for whom there are indications of neglect or abuse. These circumstances may arise from parental challenges such as mental or physical health issues, substance abuse, violence, high levels of conflict, unstable relationships, or financial difficulties.

Over a period of nine months, Family Partners provide each enrolled family between 50 and 100 sessions, delivered across three distinct phases: a startup phase, a working phase, and an ending phase. If deemed beneficial, the intervention can be extended by an additional three months. The startup phase (1–3 months) focuses on building a strong working relationship between the Family Partner and the family and establishing a critical foundation for the subsequent phases. During this phase, home visitations are more frequent, particularly at the beginning, enabling the Family Partner to understand the family’s strengths and challenges and to identify the areas where support is most needed. The working phase (3–6 months) serves as the core of the intervention. This phase shifts from practical assistance and relationship building to more active and intensive guidance. Addressing the identified challenges and supporting the family’s progress. The ending phase (6–9 months) gradually reduces the frequency of home visits. During this phase, the Family Partner helps the family transition toward greater independence and prepares them for the completion of the intervention.

The intervention is grounded in three key theoretical perspectives. First, it incorporates Bandura’s concept of self-efficacy [5], emphasizing that strengthening parental self-efficacy is fundamental for achieving positive changes within the family. Second, it applies Bronfenbrenner’s ecological theory of human development [12], which highlights the importance of a holistic approach, encouraging Family Partners to consider the family’s broader social and environmental context. Finally, the intervention is informed by Albon’s theory of potential of collaborative problem-solving [19], which focuses on identifying and developing skills the child may lack through supportive dialog and collaboration.

To address the family’s challenges across all three phases, the Family Partner and the family co-develop a working plan. This plan incorporates a measurement feedback system, including monthly well-being scoring schemes, to track progress and adapt the intervention as needed. By combining systematic parental training with practical support tailored to the family’s unique needs, the intervention provides comprehensive assistance. Additionally, the Family Partner ensures that the family is connected to other necessary services to further support their well-being.

### Study outcomes

The primary outcomes in the pilot RCT were acceptability, retention and adherence to the study. *Acceptability* was measured through qualitative interviews with caretakers in the intervention group, Family Partners delivering the intervention, as well as case workers and representatives from the Norwegian child welfare services. *Retention* refers to response rates, specifically the proportion of participants completing surveys. The success criteria required a minimum of 70% retention in both groups over at least nine months, with response rates above 70% and comparable between groups. Additionally, the three child welfare offices needed to recruit and retain at least 50% of their staff for a minimum of 18 months, with the ability to replace staff in cases of turnover. *Adherence* was assessed based on whether caretakers withdrew their written consent within 12 months of recruitment. This included tracking the timing and reasons for withdrawing, as well as whether caretakers terminated the intervention prematurely [33].

To assess the potential effects of the interventions, the secondary outcomes of the pilot trial included the following measures: parental behavior (Parenting Scale Short Form, [25]), parental stress [7], parental locus of control (Parental Control of Child's behaviour subscale, [13]), parental mental health (Warwick-Edinburgh Mental Well-being Scale, [40]), parental self-efficacy (General Perceived Self-Efficacy Scale, [37]), social support (Oslo Social Support Scale, [10]), relationship to the professional helper (Working Alliance Inventory, [20]), and family routines and service use (see [34]). As outlined in the study protocol [33], a total of 90 families was estimated to be sufficient sample size for assessing the feasibility outcomes of Family Partner.

#### Recruitment and randomization procedure

Screening for inclusion criteria was conducted by case workers at the three participating child welfare offices. When a family was referred to a child welfare office, case workers assessed their eligibility to participate in the study. If the family met the criteria, the case workers approached them to gauge their willingness to participate. Caseworkers relied on clinical judgment rather than formal screening procedures. Written consent was then obtained through an online survey tool. After obtaining consent, participants completed a baseline assessment questionnaire and were randomized in a 1:1 ratio to either the treatment or control groups. Randomization was conducted using the online survey tool “Nettskjema”, ensuring that neither participants nor staff were aware of group assignments at the time of allocation. Once the randomization was completed, researchers were notified of the participant’s group assignment and subsequently informed the staff. Participants in the intervention group received support from a Family Partner, while the participants in the control group received standard services, which varied across child welfare offices. These services consisted of counseling and parental guidance provided by the family’s caseworker. The frequency of meetings in the control group was lower than in the Family Partner intervention and generally did not include practical assistance.

#### Survey and retention strategies

Participants were asked to complete five surveys over the course of 18 months. Although the intervention often treated families as a unit, a single parent was designated to complete the surveys. This responsibility was assigned to the parent with primary custody or primary caregiving duties for the child. To encourage participation across all assessments, a series of incentives were offered: T1: respondents received a NOK 200 Universal Gift Card, valid at over 5500 locations nationwide. T2: respondents were entered into a draw to win an iPad Air 2022 (64 GB), with favorable odds explicitly stated due to a small participant pool (fewer than 50). T3 and T4: respondents entered a draw for two NOK 1500 gift cards. At T4, favorable odds were again highlighted (fewer than 40 participants). T5: upon survey completion, each respondent received a guaranteed NOK 500 gift card. Non-respondents received follow-up calls or a text message from a research assistant at T1–T4 to maximize response rates. These calls invited them to complete the survey via a link sent to their mobile device or verbally over the phone. For the final assessment, the project leaders conducted these follow-up calls.

#### Qualitative data collection

To contextualize the experiences and findings from the pilot trial, participants were recruited to participate in semi-structured interviews. Participants were selected using a combination of purposive and convenience sampling. Professionals were chosen based on their knowledge of or involvement in the intervention. Specifically, all Family Partners were invited by the researchers to participate in interviews, while caseworkers were selected by managers in child welfare services due to their familiarity with the intervention and their availability. Caretakers with experience in the intervention were approached by caseworkers or Family Partners; however, it is unclear whether any caretakers declined the invitation to participate. Interviews were conducted with a range of participants: families in the intervention group (*n* = 5), Family Partners delivering the intervention (*n* = 5), those involved in implementing the intervention (*n* = 4), leaders at various levels within the child welfare offices (*n* = 8), other relevant stakeholders, including teachers, public health nurses, workers from family welfare office, and representatives from the Norwegian Labour Welfare Administration (*n* = 5). In total, 29 semi-structured interviews were conducted, along with two focus group discussions with Family Partners. Interviews were carried out by two of the authors involved in this study and lasted between 50 and 75 min. Three different interview guides were used, outlining topics such as experiences with delivering or receiving the intervention, and experiences relating to recruitment, randomization, and screening. All interviews were recorded using a voice recorder and transcribed with the assistance of Whisper from OpenAI, Inc. (USA), on a computer for increased confidentiality and read through by a research assistant.

### Data analyses

We used descriptive and comparative statistical methods to analyze retention, adherence, and secondary outcomes. Frequencies and percentages were used to summarize response rates and retention patterns, while median and mean values was used to capture the time participants spent completing the surveys. The analysis explored differences in retention and adherence between intervention and control groups. We assessed missing data and patterns of non-response and helped evaluate the relevance or burden of survey items. The small sample size limited inferential analyses. All analyses were conducted using Stata 18.5.

### Analysis of the qualitative data

The qualitative analysis was conducted by the first and third author in MS Word, following Braun and Clarke [9] six-step guide to thematic analysis. (1) The interviews were reviewed to gain familiarity and note initial ideas. (2) Relevant features of the data were coded, focusing on the accessibility and feasibility of conducting an RCT of the Family Partner intervention. (3) The generated codes were organized into potential themes. (4) The identified themes were reviewed to ensure they accurately represented the data. (5) Themes were clearly defined and named to capture the essence of the data. (6) The final themes were used to produce the report of the results.

### Ethics

A study protocol was published prior to the pilot-RCT [33] and the trial was registered on ClinicalTrials.gov (Identifier: NCT04957394). The pilot project was reviewed and approved by the Norwegian Agency for Shared Services in Education and Research (804,402). Participants received detailed information about the study and provided written and oral consent before participation. They were also informed of their right to withdraw from the study at any time. All collected data was securely stored on encrypted servers to ensure participant confidentiality and data protection.

## Results

### Acceptability of the Family Partner intervention

Qualitative interviews consistently highlighted positive experiences with the Family Partner intervention. Across all groups interviewed—participating caretakers, Family Partners, leaders and caseworkers from the child welfare service, and other stakeholders—the majority expressed strong confidence in the intervention’s value for numerous families, as previously reported by Finne et al. [17]. In the following section, we elaborate on the perceived effectiveness of the intervention. Both Family Partners and child welfare workers, along with other collaborating agencies, emphasized the importance of an intervention like Family Partner that allows sufficient time to get to know families in a natural and calm manner. The most consistently praised aspect of Family Partner was the significance of dedicating time to build strong relationships and trust between the Family Partner and the participating families. The first three months of the intervention, devoted to relationship building, were frequently referred to as a critical success factor for fostering meaningful change within families during the later phases:

**Table:** 

“That, I feel, has really built a lot of trust in me. There has never been this kind of rush where they say,'We see that you are here, but we actually want you to be over there,'or anything like that. We have been met with understanding the whole way through all of my steps, and I think that has been absolutely crucial for me to be able to maintain trust.”	“But I also think about relationship-building in terms of the child as well. Instead of being so stressed about creating change, we can actually take the time to play with the child and just be present. And get to know each other that way, without having to have those conversations. Because that can also make them pull back: ‘Oh, they just want to talk about those things.’ But now, I can just be, and be present.’ At the same time, we can show the mother how she can be present for the child, without us having to guide her so explicitly.”	“Also, the fact that they are involved for a longer period. That’s really nice. It’s kind of reassuring to know that, yes, they will be involved for a long time, that there’s plenty of time to assess, get to know each other, work, and that you can extend the time if you see that something took longer than expected. For example, there’s a family now where we didn’t really get anywhere or felt like we had to wrap things up, because there was no reason to force any measures. But just then, right at the very end, they opened up. And then we could continue working after all. So, the fact that they’re involved over time.”
(Participant)	(Family Partner)	(Caseworker)

Over time, families became more comfortable with the Family Partner, which fostered honesty about their needs and, in turn, increased their openness and motivation to accept guidance. Several Family Partners highlighted that the time dedicated to building relationships and trust was crucial for addressing challenges that would have been difficult to tackle without this foundation. Both Family Partners and child welfare workers described the intervention as a holistic and tailored approach. By spending time with the family, the Family Partner developed a deeper understanding of the complexity of their problems. This understanding enabled the Family Partner to address multiple challenges simultaneously while prioritizing the most pressing issues. Although relationship-building is intended to occur during the initial phase of the Family Partner intervention, both Family Partners and caseworkers observed that, for some families, this process took longer. In such cases, Family Partners often reported working more intensively toward the end of the intervention, as families only began to open up and fully engage in guidance at that stage.

Most families receiving the Family Partner intervention reported positive experiences. The interviews revealed that many families felt that the intervention provided emotional support and a sense of security. The sense of security was largely attributed to the flexibility and availability of the Family Partner. Families described feeling cared for on their own terms, met with understanding, and reassured that they were on the right path. The practical assistance offered by the Family Partner was also perceived as highly supportive. As one participant explained, “Just knowing that they are there has been, I think, my greatest source of security”.

When necessary, the Family Partner connected the family with other support services and agencies within the municipality. These agencies most commonly included social services, school-based services—such as teaching and nursing—mental health services for children and adolescents, as well as educational, psychological, and family counseling services. The Family Partner often accompanied families to these meetings, providing emotional support and a sense of security. Families noted that this support was particularly valuable in facilitating communication, as the Family Partner helped them prepare for and follow up after such meetings. Stakeholders from these agencies also expressed that collaborating with the Family Partner brought them a sense of relief. The Family Partners’ deep understanding of the families’ challenges and ability to communicate their needs to the relevant agencies made it easier for these services to focus on specific areas of support in collaboration with the family.

**Table:** 

“And also, the Family Partner is a very significant help and relief for caseworkers. In terms of not having to coordinate all the interventions and attend every single meeting, you can set aside a lot of the follow-up. Instead, you just pull the strings and keep track of things yourself, as opposed to having to do everything on their own”	“Yes, I actually felt that it has been very nice because they have been very available, and I have communicated a bit with them outside of the meeting points we’ve had. When I felt the need to inform them, for example about things I’ve observed and learned, I’ve been able to communicate that further and received feedback on it. It has felt like a very flexible and dynamic collaboration, what should I say, where we have supported each other towards a common goal”	“It was quite hopeless to seek help, both privately and publicly. Having to make phone calls and attend calm meetings and such, I got pretty tired of it after a while. (…) Yes, because it's like, there are so many meetings. (…) And then I’ve had to sit and explain the child’s problem over and over again to new agencies. I kind of hit rock bottom then. But I came back up from that, and that’s when theFamily Partner understood they should start speaking up for me. (…) Then they supported me personally and in everything overall. I’ve been really satisfied with that”
(Caseworker)	(Stakeholder)	(Participant)

### Practical challenges in conducting the study

Two key challenges emerged during the interviews regarding implementation of a large-sale RCT. The first challenge related to the varying capacity of Family Partners, which was heavily influenced by the complexity of the families’ cases. When asked about how many families a Family Partner could realistically support, both Family Partners and child welfare service employees commonly noted that it was nearly impossible to determine due to the diverse needs for family follow-up. An identified strength of the intervention was the assignment of two Family Partners to each family. This arrangement was seen as an advantage, as it allowed the Family Partners to work closely together. Both Family Partners being familiar with the participating families reduced vulnerability in cases of illness or absence. Furthermore, their differing professional backgrounds enabled them to complement each other’s strengths, share experiences, and discuss challenges, ultimately enhancing the support provided to families. This collaboration was especially beneficial when working with particularly complex or demanding cases, offering both practical relief and improved outcomes.

The second major challenge in conducting a large-scale RCT concerned the ethical aspects of the recruitment process and randomization. Case workers, who were responsible for screening and recruiting participants for the pilot trial, highlighted several difficulties in this task during interviews. One key challenge was presenting the study to potential participants in a transparent yet balanced manner. Case workers needed to provide sufficient information about the study while avoiding overemphasizing the intervention, which could result in disappointment if participants were assigned to the control group. As reported in Finne et al. [17], Family Partners were disappointed when participants who seemed most likely to benefit from the intervention were assigned to the control group, which over time took an emotional toll. Likewise, caseworkers found it difficult to invest time and resources recruiting participants only to see them randomized into the control arm.

### Child welfare services as a barrier to the intervention’s reach

Reflections from the interviews revealed concerns about the potential drawbacks of situating the Family Partner intervention within the child welfare service. Several of the Family Partners questioned whether this affiliation might limit the reach of the intervention. The majority acknowledged a general skepticism towards the child welfare service, which could deter families within the target group from engaging with an intervention like Family Partner.

Among the interviewed caretakers, many expressed that accepting assistance from child welfare service made them feel particularly vulnerable. One participant shared: “(…) inviting completely unfamiliar people in, especially from the child welfare service. It does create some hesitation, it really does. (…)”. Additionally, families reported having low expectations of the support the child welfare service could provide. One family explained: “No, I didn’t have any expectations at all. I was actually a bit surprised. We didn’t quite understand what the child welfare service could help us with. We haven’t had any violence or anything like that in our family”.

Among the interviewed caretakers, few reported negative experiences with the intervention itself. However, some families had prior negative experiences with the child welfare service, which significantly heightened their skepticism toward the intervention. This skepticism often shaped their perceptions of the intervention’s utility. One participant explained: “We do have conversations too, but I don’t know, it’s like, I could just as well have gone to a psychologist if I really wanted to talk to someone. There’s nothing I need to discuss with them. It’s not the child welfare service; if I had problems, they’re not the ones I would talk to. I would have found a friend or someone else, yes. But I had that. I don’t know how they think. I don’t know what they think that setup is supposed to help me with”.

Another challenge related to the intervention’s affiliation with the child welfare service was whether participants perceived consent to participation as truly voluntary. Although the Family Partner is entirely voluntary, our data suggests that some families felt pressured or “in a pinch” when deciding to participate. One participant shared: “I was told that I should participate; I could be selected for two different things. I responded to them, and then they told me that I would have to answer the same questionnaire about improvements and provide feedback again ina few months. But then, suddenly, when the children were taken off the child welfare service, Family Partner became involved. Then they were going to be right on top of me”.

### Retention

Table 1 outlines the retention rates at each time point in the study. Participants (*n* = 45) were distributed between two groups, with 23 allocated to the control group and 22 to the intervention group. All 45 participants completed the baseline survey (T0). At the first follow-up survey (T1), retention dropped to 27 participants, representing a 60% response rate. Retention measured through survey responses continued to decline at subsequent time points: 40% (18 participants) at T2, 31% (14 participants) at T3, and 29% (13 participants) at T4. However, retention showed an improvement at the final time point (T5), with 19 participants responding, yielding a 42% response rate.

**Table 1:** Response count at each time point

Time point	Baseline (T0)	T1	T2	T3	T4	T5
Number of responses	45	27	18	14	13	19
Response rate (%)	100	60	40	31	29	42

**Fig. 1:**
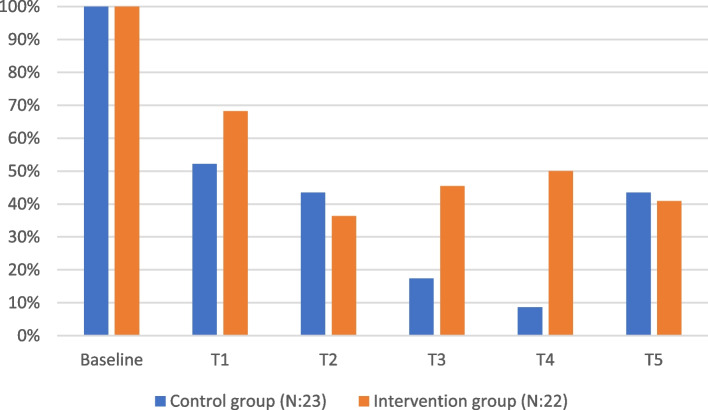
Response rate at each time point, by group

As shown in Fig. 1, response rates varied between time points and groups, with notably lower rates in the control group at T3 and T4. At T1 and T5, where all respondents received a gift card upon survey completion, response rates were higher. In contrast, at T2, participants were entered into a draw to win an iPad, and at T3 and T4, they were entered into a draw to win gift cards. As seen in Fig. 1, a higher number of participants from the control group responded at T2 compared to T3 and T4. While providing gift cards to all respondents might be the most effective strategy, it is significantly more costly when working with larger sample sizes. At each assessment, non-respondents were contacted by a project member, except at T5, where the follow-up calls were conducted personally by the project leader (second author). However, the data suggests that the role of the person conducting the follow-up calls—whether a project member or the project leader had no noticeable impact on retention rates.

Figure 1 shows the number of participants who responded at each individual time point. However, there was variation in the total number of surveys each participant completed throughout the study period. Retention rates varied among participants, reflecting differing levels of engagement with the study. Seven individuals responded only at baseline, while 14 provided responses at baseline and one additional time point. Five participants completed baseline and two follow-up surveys, and 12 responded at baseline and three follow-ups. Four participants completed baseline and four follow-up points, while notably, three individuals maintained full retention across all six measurement points (baseline and five follow-ups).

### Adherence

Adherence in this study was strong, with no participants withdrawing their consent. However, two of 45 participants opted out of further participation in the surveys, actively declining additional follow-up assessments. Qualitative data revealed several challenges related to participants’ lack of interest in completing questionnaires. Although incentives were offered at each assessment, interviews revealed that families generally showed low commitment to responding. Common reasons included fears of fraud, negative experiences with child welfare services, or simply a lack of time. One participant suggested that integrating surveys into the Family Partner sessions might improve adherence: “I’ll be completely honest; it’s just me. If there had been a way for theFamily Partner to incorporate this into one of the sessions, I had here—like setting aside 20 min for me to fill out the form—I think I might have done it”.

### Secondary outcomes

The surveys included various secondary outcomes to assess the preliminary effects of the intervention. However, the low response rate in the pilot study limits the ability to assess these effects meaningfully. At each assessment, the total number of respondents was too low to detect significant differences between the intervention and control groups. While small differences were observed, the limited sample size makes it impossible to determine whether these differences are attributable to the intervention or to random variation. Despite these limitations, the survey results provide valuable insights into the functionality of the instruments within this population, participants’ willingness to respond, and survey attrition. The results from the pilot study show that most respondents completed the survey questionnaires once they started answering, this indicates a low “respondent dropout rate” from the surveys. This applies for all six surveys. In terms of survey response time, our results show that response times for both groups decreased between T1 (median 14.5 min) and T3 (median 10.5 min), before increasing again by T5 (median 14.0 min), with mean times showing a similar trend, peaking at T5 (18.3 min). None of the scales used included a response option for “don't know/not relevant”. Few of the questions in the surveys had missing values.

## Discussion

The primary objective of this study was to assess the acceptability, retention, and adherence of Family Partner, a home-visiting intervention within the Norwegian child welfare services. Acceptability was assessed through qualitative interviews and results suggest a high level of acceptance across all groups interviewed, including participating families, Family Partners, leaders, and caseworkers in the CWS. Key components to the intervention’s success, identified through the qualitative data, included its perceived value for families, the strong relationships fostered between Family Partners and families, and its holistic approach to support. However, practical challenges emerged during the trial. Particularly, determining feasible caseloads for Family Partners administering the intervention proved difficult, given the complexity of the families’ needs. Additionally, skepticism toward the CWS, where the intervention is based, was a significant barrier for many families. This finding aligns with previous research highlighting ambiguous attitudes toward child welfare in Norway [39], and in other countries [38, 44, 46]. While this highlights an inherent challenge in conducting research within CWS, it also underscores the importance of building trust and fostering strong alliances with those recruiting families and delivering the intervention.

Adherence to the trial was high, with no participants withdrawing their consent to continue the study. However, two individuals (4.4% of the 45 participants) opted out of further participation in the surveys. In terms of retention rates, assessed through survey responses, the trial did not meet the predetermined criteria of a minimum of 70% retention in both groups over at least nine months, with survey response rates above 70% and comparable between the groups. Indeed, following the baseline assessment, the retention rate dropped to 60% and continued to decline, reaching 42% at the final assessment. Notably, the intervention group demonstrated more stable response rates, likely due to their stronger engagement with the study and closer follow-up from the Family Partner. This direct relationship may have enhanced their motivation to complete the surveys. The control group, lacking this personal connection, appeared less motivated to complete the surveys.

However, the response rate across the data collection points is considered low compared to the average online survey response rate [45]. This outcome is not surprising given the well-documented challenges of collecting longitudinal data from vulnerable and underserved populations [8]. It should be noted that we experienced high adherence to the intervention, and few missing values in the surveys, which suggest that the surveys imposed a low respondent burden, were perceived as relevant by the respondents, and were well suited to the target population.

To address the low survey retention rate, the research group implemented several strategies. These included contacting participants via phone calls and text messages and offering incentives such as gift cards or the chance to win an iPad at various stages of the study. Our data suggests that the role of the person conducting the follow-up calls—whether a project member or the project leader—had no noticeable impact on retention rates. Previous research suggests that monetary incentives positively influence response rates in online surveys, both in the general population [1, 23] and among vulnerable groups [26]. Our findings align with this research, as the highest response rate was achieved during the final follow-up, when participants were offered the largest financial incentive and personally contacted. These results suggest that monetary incentives, coupled with personalized follow-up, could be effective strategies to improve survey retention. Additionally, emerging research in the Norwegian CWS suggests that in-person assessments conducted by researchers may be a viable option for ensuring higher response rates [14]. Future trials conducted in similar settings should consider incorporating both monetary incentives and alternative assessment methods, such as in-person data collection, to optimize survey response rates.

This study does not report on the effects of the Family Partner intervention. However, the findings underscore previous research that highlights the inherent challenges of conducting RCTs in child welfare settings [17, 24, 30]. The results emphasize the importance of pilot studies in optimizing the likelihood of success before implementing large-scale RCTs. The challenges of conducting experimental research with vulnerable populations—such as families involved with child welfare services—may contribute to the limited use of evidence-based practice in the CWS [16, 17, 24, 30, 35, 36]. Our findings have outlined challenges concerning randomization of participants within CWS. Finne et al. [17] provided a detailed discussion of alternative designs for a full-scale evaluation of the Family Partner intervention. They suggested options such as block randomization to increase the likelihood of balanced group sizes, cluster randomization, or the use of pre-established allocation lists instead of individual randomization. Notably, each design has its own strengths and limitations.

Nevertheless, addressing the challenges associated with carrying out experimental research in the CWS is essential, as the consequences of child maltreatment are severe, with long-term impacts on mental health, well-being, and life outcomes [2, 4, 18, 21, 29]. Given these stakes, it is critical to study current practices to determine what works, for whom, and under what conditions. By identifying and addressing barriers to conducting rigorous research in child welfare settings, we can improve intervention strategies and better support vulnerable families.

## Limitations

This study has some limitations that warrant consideration. First, we did not meet our target of recruiting 90 families for the Family Partner study. This shortfall may be attributed to the intervention’s placement within the CWS and the inherent challenges of conducting experimental research with vulnerable populations. Consequently, the limited number of recruited families resulted in insufficient statistical power to perform robust analyses of secondary outcome measures. Another limitation of this study is that we did not collect data on the number of families approached to participate, so we are unable to present the participant flow. A key limitation of this study concerns the measurement of adherence. As outlined in the study protocol, we assessed adherence based on whether families withdrew from the intervention prematurely, including documentation of the timing and reasons for withdrawal. While this approach was pre-specified for the exploratory aims of the pilot trial, it provides only a partial view of adherence and does not capture the extent to which participants actively engaged with specific components of the intervention. Remaining in the intervention does not necessarily equate to following it as intended. In future full-scale trials, more robust and detailed fidelity measures should be implemented to allow for a systematic assessment of both adherence and the quality of intervention delivery. In conclusion, our findings highlight the acceptability of the Family Partner intervention and adherence to the trial protocol. Despite a low retention rate and specific practical challenges, the Family Partner program demonstrated its potential as a valuable home-visiting intervention to support families navigating parenting difficulties, and ultimately, that may reduce child maltreatment.

## Data Availability

Data can be made available upon reasonable request to the second author.
